# A Report upon, the Latest Achievements in the Sphere of Epilepsy, from the German of Dr. Finkelnburg, of the University of Bonn, Germany

**Published:** 1864-01

**Authors:** 


BUFFALO

### Medical and Surgical Journal.

VOL. III. JANUARY, 1864. NO. 6.

ART. I.—*A Report upon the latest Achievements in the Sphere of Epilepsy, from the German of Dr. Finkelnburg, of the University of Bonn, Germany. Translated, and with notes by* H. LASSING, M. D., *Physician and Surgeon to the Eastern Dispensary, New York.—Concluded.*

For the Buffalo Medical and Surgical Journal.

After the important precedent set by these two investigators, and probably somewhat more guided by the results of their labors than it would seem is willingly acknowledged, appeared the researches of Schroeder Van der Kolk upon the medulla oblongata, which again formed an epoch in the progress of research upon our theme. (Schröder van der Kolk, J. L. C., Bau und Funktionen d. medulla spinalis und oblongata und naechste Ursache und rationelle Behandlung der Epilepsie. Aus d Hollaendischen v. Dr. F. W. Theile. Braunschweig, 1859.) This author proclaimed thé medulla oblongata to be the seat of epilepsy, eclampsia, chorea, tetanus, globus hystericus and hydrophobia, and with a hitherto unattained precision proved the origin and anastomosis of all those motor nerves, which in the epileptic attack play the main part—i. e. facialis, accessorius, port min., trigemini, hypoglossus—as well as their organic connections with the ganglionous central points of certain lines of sensational nerves in the medulla oblongata. Besides this clearer solution of the physiological, foundational relations the same investigator brings to light an entirely new pathological, anatomical state in the medulla oblongata of epileptic patients. The observation with the naked eye of the redness of the fourth ventricle and the adjoining marrow led him to make a closer microscopical examination of the same which extended itself over fourteen inveterate cases of epilepsy.

The results of these examinations culminated in proving the existence of considerable measurable capillary ectasis with thickening of the coats and pithy albuminous infiltration of the surrounding medullary substance. In those cases who during the attack regularly bit their tongue, these changes were mostly found in the course of the hypoglossal nerve and the olivaris, in the others more or less exclusively in the course of the vagus, a difference which seemed of such importance to the observer as to lead him to divide epilepsy according to the fact whether or not it was accompanied by biting of the tongue. The degree of capillary enlargement compared with the normal medulla oblongata varied in these fourteen cases from two-fold to the four-fold. In conjunction with this he found enlargement of the vessels in the brain, albuminous intercellular fluid and atrophied ganglion-cells—particularly in the corticular substance—with accompanying idiocy and imbecility.

It it not a little to the credit of Schröder v. der K., to draw the inference, of his discoveries with a most delicate modesty, although he could not completely withstand the temptation of a one-sided explanation. In the enlargement of the vessels he does not see a cause, but an effect of epilepsy which nevertheless in its turn, acting reflex, increases the obstinacy of the disease and enlarges the extent of sympathetic organic complications. He holds that to produce epilepsy it does not require any disorganization, no decisive change of tissue, only a heightened excitability, which is mostly accompanied by a re-inforced flow of blood and an increased change of substance. The morbid excitement can show itself upon the medulla oblongata if arising from the peripheral parts of the body through the spinal nerves. if from the bowels through the vagus or sympatheticus. (Kussmaul observed in a rabbit, whose carotis had been ligated, that every galvanic irritation upon the upper severed part of the sympatheticus produced convulsions, which upon the intermission of the irritation, ceased.) Again, a morbid admixture of blood can produce the highest state of excitement in the ganglion cells of the reflex organ. The loss of sensation Schröder van der K., after the precedent of Kussmaul and Tenner, with probability ascribes as a consequence of spasmodic contraction of the cerebral arteries, but again in opposition to these observers claims an active arterial congestion in the medulla oblongata itself, at the beginning of the attack. This theory lacks a sufficient foundation, as the appearances in life he describes as well as the capillary ectasis discovered in the cadaver can be fully accounted for by the much more probable cephalic venous congestion existing during the second and third stage of every well developed epileptic paroxysm.

The rational treatment of epilepsy according to this writer requires: (*a*) Reduction of the heightened excitability of the medulla oblongata, and if necessary decrease of the strong sanguinous afflux to these parts. (*b*) If possible removal of the remote cause which through their influence upon the medulla oblongata sustain the excitability to the pathological reflex action. Among these remote causes, he considers as the most frequent, an increased nervous activity of the bowels or generative organs, after this excitement of the brain of a psychical nature. The most of the so-called specifics, such as the flor. zinci, lunar caustic, artmesia, and others seem to act by reducing the heightened excitability of the bowels. A direct action upon the medulla oblongata they do not have, and therefore this must at the same time be acted upon and its heightened excitability be allayed by *dispersive* remedies, as by seton in the nape of the neck, incisions in the scalp; in plethoric patients leeches, or cupping. In inveterate cases of epilepsy with idiocy, he met with success—in *one* case with radical cure (?)—from the repeated application of the actual cautery to the scalp.

Although the investigators already named, succeeded in elucidating the biological results of epilepsy and its histological products, that is the middle and end link of the pathological change, the desiderata most important to physicians, the elucidation of the commencing link of this chain, remained unattained. The task remained to grasp that unknown something, which was anatomically as well as chemically invulnerable, and yet must form the real and specific basis of epilepsy, and according to its appropriate relations limit it within the smallest lines possible, and thereby approximate to an understanding of its real nature. This object which in England was pre-eminently pursued by Sieveking, in Germany after Romberg's precedent seemed to animate Wittmaack, who sought by a scientific treatise, to penetrate the theories upon neuroses, and to vindicate its original separation as against the influence upon it by modern pathological anatomy and chemistry, (Wittmaack Theod. The intermittent, chronic cerebral spasms, epilepsia, pathologically and therapeutically viewed.) The real pathological foundation, the primogenetical basis of the epileptic affection, he says, is unmistakably owing to peculiar constitutional disposition of the nervous system. The postulate of such a specific disposition arises from the fact that all the causes of epilepsy usually enumerated, appear much oftener *without* than *with* it, and their influence or power is that *opportune* moment, which find a chance of invasion only where the balance of the central function regulators are already disturbed. The somatic type of the "to epilepsy disposed," W. found mostly in an *easy, nervous irritability upon a weak organic basis.* There are two large classes of individuals who, in particular, offer a broad basis to this epileptic predisposition. The first of these includes those lymphatic erethic constitutions to which the well known terminus technicus of the physiological school usually ascribes an irritable weakness with feeble power of resistance, i. e. great vulnerability of the nervous action—individuals of a fine gracile organization with slight plastic energy, often with a well expressed tendency to scrofulous and tuberculous anomalies. Particularly the female sex and those between ten and twenty years of age, furnish frequent epileptic examples of this class. Menstrual anomalies accompany this upon the same basis, and are erroneously taken up as independent causes.

A second class of those disposed to epilepsy is formed, according to Wittmaack, by individuals of an apparently robust habit, in part plethoric and corpulent, formerly so-called leuco-phlegmatic, bloated subjects to whom the former physiological school ascribed a torpid irritability, heightened excitability with a weak power of reaction.

It can be seen how this author seeks after precise categories to limit those peculiar abnormalities of the constitution *preceding manifested epilepsy*, to grasp within definite classes its symptoms. If with unsatisfactory results, it is still his merit to have brought this great practical question again in the foreground, and to have pointed the way to its solution. In regard to the psychical immediate causes of epilepsy, W. expresses himself to the effect that they most generally bring their influences to bear in the shape of fright and fear, and then by means of an overpowering of the cerebral powers of reaction produce what might be called a psychical paralysis—offering a momentary open field to anomalous reflex action. These moments where a predisposition exists can be occupied by epileptic convulsions. He considers plethora and anæmia as equivalent causing states to awaken the predisposition; yet the latter appears to occur oftener than the former.

His pathological views correspond with his views of *indications.* These are characterized by caution in the use of the usual and even the most celebrated anti-epileptic remedies and by the preponderating endeavors to make the *entire disease* "the alienated nervous system with the organic constitutional alienations," the subjects of cure. For example, he is very definite in his treatment in those frequently occurring cases caused by the so-called nervous erethism, that is, that temperament resting upon a great excitability without an equal base for an equivalent natural reaction. Here the application of powerful remedial agents are not to be thought of. The most important means are a strict regulation of the diet, occupation, (particularly mental,) exercise and quiet, consequently the success of a residence in the country, the milk, whey and grape cures.

Another kind of influence upon the system is necessary in torpid subjects; it can be more powerful, exciting, and in part withholding. For this reason, besides a somewhat stimulating diet, cutaneous stimulants, particularly that of cold come in properly here. W. says, "one cannot act more efficiently upon the cerebral energy, through any other organ than through the skin, with its numerous nervous plexuses, and I therefore know of no remedy which answers the required purpose as well as sea-baths, cold ablutions, or the cold affusions already celebrated by *Celsus*."

Of dispersive remedies, W. found fontanelles and setons the most effectual, but they must be continued a long time. Systematic gymnastics he advised in those cases where chronic abdominal troubles formed the basis of the epileptic attack.

The several results of labors upon the subject under our hands found a splendid conclusion in the work of Reynolds, (J. Russell Reynolds, Epilepsy, its symptoms, treatment and relations to the chronic convulsive diseases. London, 1861,) who combined a masterly review of all foreign researches with the results of a ripe personal experience, and independent style. Reynolds insists upon a strict separation of pure or idiopathic epilepsy—that is, those cases in which aside from the known symptomatic complications of the disease no other disturbances are observed, from deuteropathic or symptomatic epilepsy which are referable to disease of specific organs, external to the nervous centres, such as of the heart for example. Only the study of those of the idiopathic form can lead to clear results upon the nature and action of epilepsy. The two characteristics in the complication of symptoms are chlonic spasm and insensibility. As the point of origin of the first Marshall Hall, Brown Sequard, and Schroeder von der Kolk, point positively to the medulla oblongata, and the upper portion of the med. spinalis. The second characteristic symptom, the pause of sensation, depends physiologically upon a specific change in the organ of sensation, the large cerebral hemispheres. Congestion of the brain, according to the old school, is the commencing link of the entire epileptic appearance, and according to Marshall Hall it forms the connecting link between the spasm and insensibility. From the primarily affected medulla oblongata the irritation is conveyed to the vasamotor nerve of the cerebral arteries, and by spastic contraction of the latter produces insensibility.

As we can accordingly consider the seat of the disease as pretty definitely ascertained, the second question arises, what the nature of the change in the affected organ in epileptic patients, the medulla oblongata, is. Anatomical researches, among which Reynolds considers those of Schroeder von der Kolk the best, have shown that the changed function after a certain duration, always traceably is followed by a change in the state of assimilation, but that these changes in the assimilation are secondary, hence form no basis for the solving of the nature of the disease. Primarily it only shows the alienated function, regarding which the further question arises, whether it is changed in quality or in power. The medulla oblongata performs the specific office to change adducted sensatorial impressions into motor momentums, to reflect them upon the motor nerves. The main elements of a convulsive attack may be recognized in the normal state, and as portions of normal organic functions. The insensibility during sleep is a normal appearance, equally so the continuance of involuntary reflex muscular movement during sleep. *How* insensibility during sleep arises, has not as yet been positively ascertained, and the theory of Laycock is the most feasible, that "the conduction of the excitants received by the sensatorial nerves to the organs of sense and volition through changes in the medulla oblongata are impeded." On the other hand we see hourly changes not only under the influence of the will, but also of emotion, sensation and visceral impressions, different muscular actions and changes in the circulation. Respiration has its centre in the medulla oblongata; easy and quiet where there is no source of disturbance, mental emotion creates sobs and sighs, fatigue makes it gaping and irregular, impeded and stagnated by fear or violent physical exercise. The jaw and hands are closed involuntarily by anger, the face by excitement becomes distorted, blushes with shame and pales with fear. Thus we meet the phenomena of the spasm singly in daily life, as appertaining part of our normal activity. Reynolds therefore concludes that we have no ground upon which we can consider these changes in function peculiar to epilepsy as those of modality. They appear ill-timed, combined reversely and particularly changed in violence, yet we can see no new element of activity, and no change in the functional modality of the natural powers. That this gradual change of function shows an exaltation and not a reduction, Reynold's proves contrary to Radcliffe's theories upon physiological grounds.

For the origin of the epileptic exaltation of action in the medulla oblongata he gives us five categories of causes.

1st.—Hereditary acquisition which he found in one-third of his cases, and as he says does not in any way clear up the orign of the disease.

2d.—Deep-seated, general change of assimilating power of a physiological or pathological nature in which the nerve centres participate; hence the prevalence of the attacks during dentition, development of puberty, and pregnancy, further the development of the disease during pneumonia or an rheumatic fever.

3d.—Excessive external stimulants, physical as well as psychical.

4th.—Accidental or habitual supervention of a precedent analogous to epilepsy. Hooping-cough, excessive coitus or masturbation can lead to epilepsy, also the spasmodic laugh produced by tickling of the soles.

5th.—Organic lesions in any part of the central-nervous system, tumors, chronic meningitis, softening of the brain, neuroma, etc., can produce epilepsy through conduction of the irritation they produce upon the medulla oblongata. The nearer a tumor is located to this the more of a source it furnishes for the origin of epilepsy.

Combinations of the here detailed momentums of origin form the rule, exclusive action of one of these, the exception.

The epileptic paroxysm Reynolds divides into three stages.

*a.* Tonic spasm with insensibility, (cerebral anæmia.)

*b.* Chlonic spasm with continued insensibility, (cerebral hyperæmia.)

*c.* The gradual returning volition of motion, through a stage of change of stupor, (cerebral prostration.)

From a therapeutic point of view Reynolds divides epileptics into three classes.

1.—That class where the general bodily state of health with the exception of the paroxysms remains intact, and in these the least success is to be hoped for. Diet, regimen and counter-irritation are the only means which sometimes produce an improvement.

2.—Where anæmia or other symptoms of a debilitated constitution show themselves, iron, quinine and tonics generally, stimulants even, besides a corresponding diet and careful regards of the functions of digestion are indicated.

3.—Increased irritability of the nerve-centres outside of the attacks, demand the use of sedatives, like opium, hyosciamus, belladonna, etc. Reynolds never saw any satisfactory results from the use of the much lauded specifics, particularly of the metalic kind.

Among the isolated contributions to the casuistry of epilepsy, we find worthy of mention two cases observed and explicitly described by Schnee, (Schnee, Carl Emil, Zwei Fälle als Beitrag zur Kentniss der Reflex-Epilepsie, mit mikroskop. Untersuch d Errengenden Nervenpartie. *Zurich*, 1861.)

The questions whether syphilis and epilepsy are related, is answered in the affirmative, based upon observations made by J. F. Duncan, (Dublin Journal, xxxv, p. 48, Feb. 1863,) and Jackson, (Med. Times & Gazette. June 22, 1861.) The first describes three cases of affection of the nerve-centres by syphilis. Jackson's observations are mostly based upon autopsies. Neither of these gentlemen, however, give us sufficiently satisfactory evidence that there did not co-exist any disease of the brain independent of the syphilis.

The relation of the assymmetry of the cerebral hemispheres to epilepsy as investigated by Dr. L. Duchense, (Gaz. hebd. viii, 1861,) showed but a slight difference, as a rule, in the weight of the hemispheres.

The majority of the isolated communications upon epilepsy originated from the question of *treatment* of the disease, without materially advancing our knowledge of the same.

Rigodin (Rev. de Ther. Med. Chirurg. p. 566, Nov. 1858,) speaks highly of the combination of ext. belladonna with rad valer, as effectual in simple inorganic epilepsy, and as a new remedy in obstinate cases he recommends expatriation, particularly a somewhat prolonged residence in the tropics. Several old cases in his practice recovered, he claims, upon a removal from France to New Orleans and Central America.

Cornell (Charleston Medical Journal xviii, May, 1858,) made extensive use of digitalis, which is an old Irish remedy against epilepsy. Corrigan saw many cures performed with this remedy in the hands of old women. Ordinarily ℥iv of the fresh leaves are macerated and made into a decoction with Oj boiling water, and of this every two days ℥iv are given with an addition of the radix polyp. or every three hours, until vomiting ensues. The reputation made by these empyrical cures induced Cornell to give digitalis to his patients in carefully increased doses until the pulse became reduced to 45–50, and to keep the patient so for four months. He claims to have achieved a perfect cure in several cases, in all an amelioration and longer duration of the intervals. The cumulative effects of the remedy became so apparent however in several cases that Cornell advises against its use excepting where the remedy is taken under the constant supervision of the physician.

Of the efficiency of the setons in the neck, so highly extolled by Schröder von der Kolk, Goerds (Deutche Klenik 49, 1860,) gives an example. It was a case complicated with manial delirium of a robust girl aged seventeen. After being twice cured by a long continued seton, she was permanently cured by a protracted suppuration kept up after a blister to the nape of the neck.

General blood-letting had also its advocates. Bosmorin (Gaz. des Hôp. 19, 2861,) claims to have cured two children by a venesection in the arm made at the beginning of a paroxysm.

To the study of the forensic of epilepsy, we have nothing new of any importance, and cannot expect any. How important and at the same time how difficult practically it is to decide not only upon the imputableness, but as well upon the powers of volition in epilepsy regarding a given period of time, the following apropos case wiil show. It is from the Gaz. des Hôp, 125, 1861:

A shoemaker, aged 20, for several years subject to epilepsy, followed latterly by manial excitements, without however leaving any psychical alienation in the intervals, on the day before his wedding, was attacked with a violent headache, which he knew was a premonitory symptom of epilepsy. As he had always been relieved by being bled, he asked to be bled now, but had to wait until the succeeding day before he found a competent person to do it, and had it done a few hours before the ceremony, but without any relief. During the ceremony he was downcast and still, did not utter a single word besides the requisite "yes." He had hardly left the church when the headache increased to such an extent that he had to retire to bed in a room adjoining the one in which his guests were assembled. A violent paroxysm of epilepsy ensued, and soon became mania. He ran out naked among the guests, and furiously knocked down a lady and killed his father-in-law by stabbing him with a knife. Upon return to consciousness, three days subsequently, he remembered the wedding ceremony, and believed himself to have slept after that. Upon application of his bride's family the marriage was declared void by the court, because that the patient at the time of the marriage ceremony was not fully sane, and consequently incompetent to make a valid declaration of his will.

Notes by the Translator.—In translating this very interesting paper upon Epilepsy, I believe I have given a more extensive circulation to ideas tending to produce comment, research, and perhaps may obtain for this disease that attention from the profession which is necessary to our more perfect knowledge of it. I do not by any means share in the ideas of Dr. Finkelnburg, nor do I endorse his criticisms and encomiums upon the labors of those whom he quotes. I have only translated his paper and left all remarks which I have to make to be uttered in these notes. To any one acquainted with German medical nomenclature I need not make any explanation, for some of the somewhat unusual language used in the translation, and all I need to say in explanation to any one that German is a very difficult language to translate medical papers from, particularly for one like myself, who is not a German.

The writer, whom I translate from, does not seem to have quoted much from American medical literature, hence I am compelled to add some few cases and remarks from some of our American medical periodicals, which I think will add to the value of the paper and the light we have upon the subject, but in the first place I will add a paragraph which seems to go the rounds of our daily papers, and apparently emanates from a European source. I give it without comment, letting the reader draw his own deductions:

"Something for Health—Important Medical Discovery.—A London letter says:

'A great discovery is just now engaging the attention of the scientific and medical world. Few English names are more familiar to Americans than that of Dr. John Chapman, once the leading publisher of heretical books, now editor of the *Westminster*, and always a devotee of science and medicine. This Dr. Chapman has been for years engaged in studies and experiments connected with the nervous system alone, with Dr. Brown-Sequard and Claude Bernard of Paris. For the past year he has been proving a tremendous discovery—namely, the cure of epilepsy, and many diseases hitherto deemed incurable, by means of the external application of ice and hot water, in India-rubber bags, at various parts of the spinal cord, acting thus upon the sympathetic nerve, and through it upon the most important and vital regions of the body. Many eminent physicians have accompanied Dr. Chapman to see the marvels which he had wrought upon patients who had long ago despaired of health. Many of the worst and most inveterate feminine diseases have yielded to the new cure. The treatment is as simple as it is grand. Any one who is troubled by the pressure of blood on the brain will find that, by holding a bag of ice on the nape of the neck ten minutes, an equable flow of blood can be secured. Those who are troubled with habitual cold may find relief by applying ice to the small of the back in the lumbar region. It is hard to estimate the importance of this discovery, which will ere long be ranked by the side of that of Jenner. Seven hospitals are already under Dr. Chapman's practice, and, as yet, no one can bring forward an instance of failure.' "

Again, I must mention a case as described to me by Prof. Carnochan of this city. About the time of Marshall Hall's visit to this country a case of epilepsy came under Prof. Carnochan's observation, Marshall Hall being present enthusiastically advised tracheotomy, which the Professor performed. The patient, previous to the operation, had had on an average, one paroxysm every day, and the operation was only expected to palliate, not to cure. Patient had an unusually large trachea, so much so that an instrument of a size large enough to fit in, as a canula, had to be made. The canula remained in the wound about six months; the paroxysms became lighter and less frequent, the intervals were longer, and at one time there was no paroxysm in six weeks. Upon removal of the canula a paroxysm ensued which ended fatally.

During the last three years I have had in private and dispensary practice forty-three cases of epilepsy under treatment of which I have kept a record; of these I will mention a few, and give the cause and history of the balance in a more condensed form.

Case 1.—A boy, 16 years of age, probable cause masturbation; paroxysms nearly every day; recovered upon the continued application of cantharidal collodion to the penis.

Case 2.—A young lady, aged 22 years; had epileptic attacks about the period when she should have menstruated; had amenorrhœa; upon reproducing regular menstruation, recovered.

Case 3.—A woman, aged 41 years, had had epileptic attacks for two years, averaging three a week, but at irregular intervals. Upon examination found a fibrous tumor in the cervical region pressing upon the spinal cord there; upon removal of which the paroxysms ceased.

Case 4.—A young woman, aged 25 years, of an anæmic appearance, but who had no symptoms of any other disease had epileptic paroxysms twice and three times daily. Upon administration of chalybeates they ceased.

Case 5.—A boy, aged 11 years, with epileptic attacks three or four times a week, recovered upon being removed from school and hard study.

Case 6.—A milkman, aged 37 years, who was in the habit of carrying two cans of milk upon a yoke fastened around his neck, and the weight of which principally rested upon the cervical vertebræ, had several attacks a week. Upon ceasing to carry the loaded yoke, rapidly recovered.

Cases 7, 8, 9, 10 and 11, were females where epilepsy was evidently the result of irregular menstruation. Upon the removal of which the epileptic paroxysms ceased.

Case 12, was that of an old lady who, during an attack of tonsilitis, swallowed a leech. She became affected with hysteria and epilepsy. Upon the administration of powerful purgatives and the introduction of *another* leech into her stool, (the one swallowed having been passed without her knowledge,) hysteria and epilepsy both ceased.

Cases 13, 14, 15, 16, 17, 18, 19, 20 and 21, were produced by masturbation, and were treated with epispastics. Six recovered entirely; two relapsed upon resuming the masturbation, and one died during a paroxysm.

Case 22, was dependent upon an irritation produced by an uterine polypus. Upon removal of which she recovered.

Cases 23, 24, 25, 26, 27, 28, 29, 30, 31, 32 and 33, no cause could be found, and patients all passed from my observation without benefit.

Cases 34, 35 and 36, died while under treatment.

Cases 37 and 38, no cause could be found, but recovered by the application of counter-irritation by moxa and setons along the spine.

Case 39, no cause ascertained. Recovered by affecting the general system with repeated emetics and mercurials.

Cases 40, 41, 42 and 43, were somewhat improved when first put under treatment, but soon relapsed and passed from under my treatment.

I copy the following from the Philadelphia Med. & Surg. Reporter, emanating from the pen of Dr. W. N. Cote of Paris:

"EPILEPSY.—It would be no easy task to enumerate all the remedies which have been employed against that obstinate and well nigh incurable disease known as epilepsy or falling sickness. The treatment should vary, according to the cause which occasions the disease. When it is sympathetic and arises from worms, anthelmintics ought to be employed. In some cases of epileptic fits, the oil of turpentine, in doses of from half an ounce to one ounce, has been found a very useful medicine. When teething is the exciting cause of the disease, the inflamed part of the gum should be deeply scarified, the body being kept open with laxative mediicines, and the feet bathed in warm water. If the epileptic paroxysms seem to be owing to disordered digestion, the contents of the stomach should be evacuated by an emetic, consisting of a solution of the sulphate of zinc in an aqueous infusion of ipecacuanha, the dose to vary according to the age of the patient, and the different degrees of irritability of the stomach, and so on. Bleeding has been found of some utility in cases where the predisposition to epileptic fits has arisen from a plethoric state of the system, or from a turgescence in the vessels of the head. In those cases, besides bleeding, an abstemious diet and proper exercise, and issues between the scapulæ, or a seton in the neck, may be recommended. The insertion of a seton in the neck has sometimes been attended with considerable relief when, from frequent paroxysms, a morbid condition of the encephalon had prevailed. In those cases, digitalis has also been found serviceable ; but to produce a permanent effect, the constitution must be kept under its influence for some weeks, by giving from half a grain to one grain of the powder, or from fifteen to thirty drops of the tincture, three or four times a day. Anti-spasmodics, such as valerian, castor, musk, ether, oil of amber, oleum cajeputae, arnica montana, belladonna, hyoscyamus, and opium, have been employed with more or less success. Electricity has been tried, but there are few cases on record which have been benefited by that agent. The younger the patient and more recent the complaint, the greater will be the chance of the electric current being of service.

Hitherto no specific has been found against epilepsy. Dr. Herpin, formerly of Geneva, and now practicing in this city, extols the virtues of the white oxyde of zinc in this disease. It may be administered according to the following formulas:

℞ Zinci oxydi, - - - - gr. xij.
Pulv. cinnam. comp., - - - gr. xv.
Cinchonæ, - - - - ʒj. M.
Ft in chartul., xij divide, quarum unam sumat ter in die.

*Vel,*

℞ Zinci oxydi, - - - gr. xxiv.

Extract gentian, - - - ʒss.

Syrupi, - - - - - q. s. M.

Ft. massa, et in pil., xij div.

Two may be taken morning and evening, with one ounce and a half of a decoction of Peruvian bark.

Drs. Trousseau and Bretonneau administer, with some degree of success, pills composed of 0.01 of extract of belladonna, and 0.01 of the powder of belladonna."

In the "Medical Times and Gazette" for Nov. 22, 1862, I find an article upon the treatment of epilepsy by belladonna, from the pen of Dr. J. S. Ramskill, Assistant Physician to the London Hospital and Physician to the Hospital for Epilepsy and Paralysis. He says that we must not always look for immediate and palpable beneficial results; even that we must expect the complaint for the first three or four weeks after taking it to grow worse, but that after six or eight weeks, if any amelioration occurs, it will be decided and progressive. At first the dose should be very small, and gradually augmented until the pupil shows signs of its action, and the patient complains of both alterations in sight and dryness of throat. Having obtained this result and maintained it for some time, the dose may be gradually diminished; but its effect upon the eye and throat are not to be so diminished as to become imperceptible to the patient, but only so far lessened as to cease causing absolute discomfort. The other toxic effects of belladonna are entirely uncalled for. Dr. R. prefers giving the drug in an eighth of a grain dose, three times a day, or only twice daily for a week, then one-fourth of a grain for fourteen days; a third for the next fourteen, at which time its physiological action will in most cases be manifested. He thinks it wise to halt at this dose for two or three months, slightly increasing the dose if the patient shows diminished susceptibility to its effects, decreasing if the reverse happens, and then gradually dropping it to the quantity first administered. This writer also gives us some very interesting ideas regarding the nature of the action of belladonna upon the system, but for these I must refer the reader to the original paper.

I have looked further, but do not find anything excepting that which is available to every man, tending to enlighten us further upon our subject, hence I leave it thus abruptly, hoping soon to be followed in this direction by some other observer of epilepsy.

